# Biotechnological valorization of rice waste from *Trichoderma* production through the development of a streptomyces-based biological soil conditioner

**DOI:** 10.1007/s42770-026-02039-y

**Published:** 2026-09-09

**Authors:** Natacha Melo, Mateus Torres Nazari, Vera Analise Schommer, Valdecir Ferrari, Carlos Alberto Mendes Moraes, Regina Célia Espinosa Modolo, Fernando Joel Scariot, Ana Paula Longaray Delamare, Feliciane Andrade Brehm

**Affiliations:** 1 Environmental Engineering, University of the Sinos River Valley (UNISINOS), São Leopoldo, RS Brazil; 2 Civil and Environmental Engineering (PPGEng), University of Passo Fundo (UPF), Passo Fundo, RS Brazil; 3 Mining, Metallurgical and Materials Engineering (PPGE3M), Federal University of Rio Grande do Sul (UFRGS), Porto Alegre, RS Brazil; 4 Characterization Center (NucMat), Civil Engineering Graduate Program, Unisinos University, São Leopoldo, RS Brazil; 5 Characterization Center (NucMat), Unisinos University, São Leopoldo, RS Brazil; 6 Enology and Applied Microbiology Laboratory, University of Caxias do Sul, Caxias do Sul, RS Brazil; 7 Unisinos University, Avenida Unisinos, 950, Cristo Rei, CEP 93022-750, CEP: 99052-900, Zip Code 611 São Leopoldo, RS Brazil

**Keywords:** Waste valorization, Agricultural bioinputs, Actinobacteria, *Trichoderma asperelloides*, Circular economy

## Abstract

Sustainable soil management is vital for maintaining agricultural productivity while mitigating the environmental impacts of intensive pesticide use. This study evaluated the use of rice waste generated during *Trichoderma asperelloides* production as a substrate for the cultivation of *Streptomyces* sp. BEI-18 A through solid-state fermentation to obtain a biological soil conditioner. The rice waste from *Trichoderma* production (RWTP) underwent solid-state fermentation (SSF) for seven days, and the product was characterized according to the Brazilian Normative Instruction No. 35/2006 for soil conditioners and amendments. Biological analyses included colony-forming unit (CFU) counts, enzymatic activity (β-glucosidase and arylsulfatase), and respirometry. The conditioner was also applied to soil at different rates in maize (*Zea mays* L.) pot experiments to assess its effects on soil biological parameters. The application of the biological soil conditioner increased microbial abundance, enzymatic activity, and CO₂ release compared to the control, increasing β-glucosidase classification from moderate to adequate. Although physicochemical parameters did not meet the criteria for physical or chemical classification, the material met the requirements for classification as a biological soil conditioner. The results indicate that rice waste from *T. asperelloides* production can be reused as a substrate for producing bioinputs capable of enhancing soil biological activity.

**Graphical abstract:**

**Figure:**
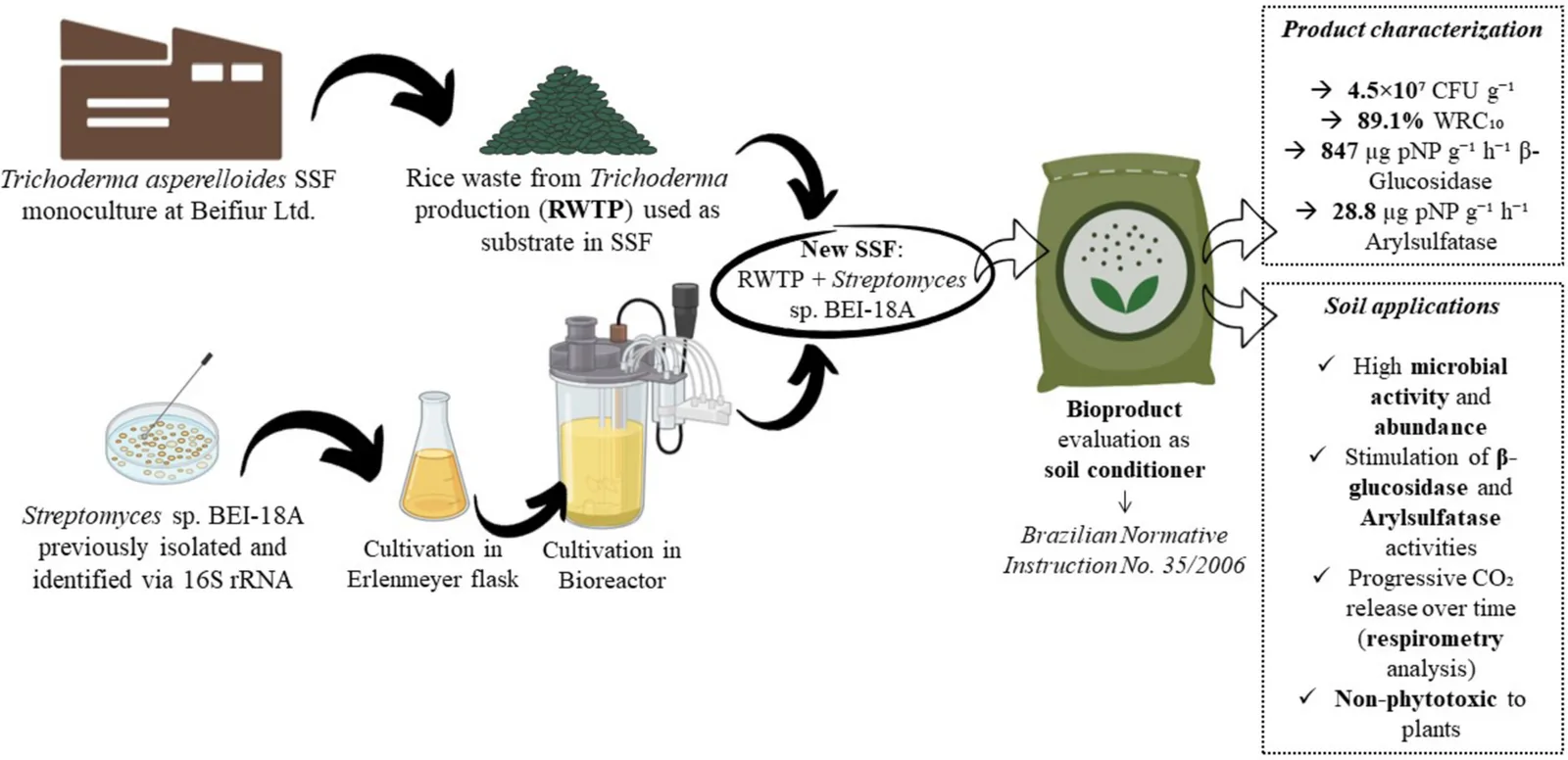

## Highlights

Rice waste from *Trichoderma* production was reused as substrate for *Streptomyces* growth.

Solid-state fermentation produced a *Streptomyces*-based biological soil conditioner.

The biological soil conditioner enhanced microbial activity, β-glucosidase, and CO₂ release in soil.

Valorization strategy promotes circular economy and supports UN SDGs 2, 9, 12 and 15.

## Introduction

The growing global demand for food, driven by population expansion, has intensified agricultural systems, increasing reliance on agrochemicals to maximize productivity and minimize losses [1–2]. Brazil is among the world’s largest consumers of pesticides, reinforcing concerns regarding environmental and human health impacts [3]. As a result, pesticides have become a central component of modern agriculture, despite their well-documented environmental impacts, including soil degradation, biodiversity loss, and contamination of soil, water, and air [1, 4–9]. In Brazil, the extensive use of pesticides is particularly concerning due to the large number of registered products containing active ingredients classified as hazardous, including substances associated with toxic effects on human health and environmental contamination [10].

An alternative to pesticides is the use of bioinputs, derived from plant, animal, or microbial sources, with emphasis on formulations based on beneficial microorganisms such as fungi, bacteria, actinobacteria, and yeasts, as well as their secondary metabolites [11]. Bioinputs are generally associated with lower toxicity and higher biodegradability, significantly reducing environmental impacts and risks to human health [7, 12–14]. In addition, they improve nutrient uptake, plant growth, and tolerance to biotic and abiotic stresses [15–20].

Among these microorganisms, the genera *Trichoderma* and *Streptomyces* are widely recognized for their agricultural applications. The genus *Trichoderma* is commonly associated with biological control, plant growth promotion, nutrient uptake enhancement, and organic matter decomposition [21–26]. The genus *Streptomyces*, one of the main representatives of the phylum Actinobacteria [27], is also known for its medical importance due to its ability to produce secondary metabolites [28–29]. In addition, the potential of *Streptomyces* in agricultural and environmental applications has increasingly attracted attention due to its association with the degradation of complex organic materials, nutrient cycling, enzymatic activity, and plant growth promotion, contributing to soil biological activity and fertility [29, 30–38].

Due to these characteristics, both genera show potential for application in agricultural bioinputs. Among these, biological soil conditioners differ from biofertilizers, biopesticides, and bioinoculants in terms of their primary purpose. Rather than focusing on nutrient supply, microbial establishment, or pest control, biological soil conditioners are intended to improve the physical, physicochemical, or biological properties of the soil. According to the Brazilian National Bioinputs Program, these products are defined as “products, processes, or technologies that promote the improvement of the physical, physicochemical, or biological activity properties of the soil” [39]. In Brazil, the classification and regulatory criteria for soil conditioners are further established by Normative Instruction SDA No. 35/2006 issued by the Ministry of Agriculture and Livestock (MAPA) [40].

The production of these bioinputs generally involves fermentative processes, such as solid-state fermentation (SSF), which allows the use of agro-industrial residues as substrates [41–44]. Although this approach is economically and environmentally advantageous, SSF generates residual solid materials that require appropriate management [42, 45–47]. The reuse of these residues in secondary processes represents a strategy to reduce waste and improve resource efficiency [13, 48].

In this context, rice waste generated during the production of *Trichoderma asperelloides* (RWTP) represents a potential substrate for the cultivation of other beneficial microorganisms. However, it remains unclear whether this residual rice substrate can support the growth of actinobacteria through solid-state fermentation and generate a biologically active soil conditioner. Therefore, this study evaluates the use of RWTP as a substrate for the cultivation of *Streptomyces* sp. BEI-18 A through solid-state fermentation, aiming to obtain a biological soil conditioner and to assess its physicochemical and biological properties in accordance with Brazilian regulations.

## Materials and methods

**Fig. 1:**
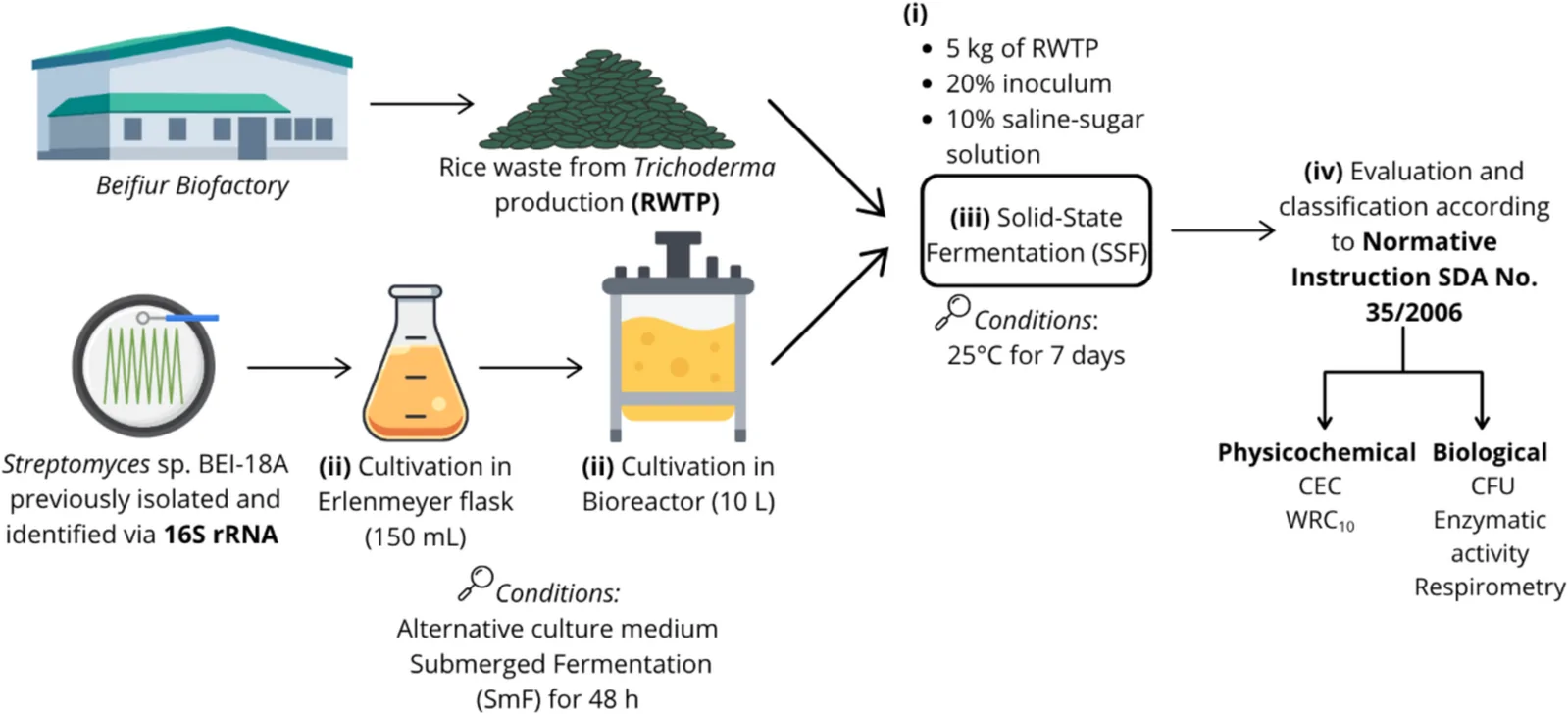
Flowchart of the process for the production of the potential biological soil conditioner

The study was conducted at the biofactory of Beifiur Ltda (Garibaldi/RS, Brazil), a company engaged in the production of organic and organomineral fertilizers, agricultural bioinputs, and in the treatment of by-products from grape processing through composting. The experimental program for the development of a potential biological soil conditioner based on the actinobacterium *Streptomyces* sp. BEI-18 A cultivated in RWTP was conducted according to the flowchart presented in Fig. 1, consisting of: (i) preparation of RWTP; (ii) cultivation of the actinobacterium at laboratory scale and in a bioreactor; (iii) application of *Streptomyces* sp. BEI-18 A as an inoculum in SSF; and (iv) physicochemical and biological analyses to classify the product as a soil conditioner, in accordance with Normative Instruction SDA No. 35/2006 [40].

### Generation of rice waste from *Trichoderma asperelloides* production (RWTP)

RWTP is generated from the production of the fungus *Trichoderma asperelloides*. The fungus is cultivated in SSF, with rice serving as the main substrate in this process. After fermentation, the rice colonized by the fungus is sieved to separate the conidia (the target product), resulting in the generation of the rice waste.

The RWTP was used without sterilization, drying, milling, or additional processing prior to inoculation, in order to preserve its original characteristics as an agro-industrial residue. Therefore, residual *Trichoderma* spores and fungal structures may still have been present in the material after conidia separation.

### Isolation and identification of the actinobacterium *Streptomyces* sp. BEI-18 A

The actinobacterial strain used in this study was isolated from samples collected directly from the composting piles at the company, as described by Nazari et al. [49]. The BEI-18A isolate was identified through 16S rRNA sequencing analysis. Total DNA was extracted and amplified by PCR using specific 16S rRNA primers (pA (5’AGAGTTTGATCTGGCTCAG-3’) and 1492r (5’TAC1GGTACCTTGTTACGACTT-3’)). The amplified product was sequenced on a genetic analyzer (AB 3500 Genetic Analyzer, Applied Biosystems). Nucleotide sequences were compared using the BLAST algorithm (Basic Local Alignment Search Tool) with the sequences deposited in the NCBI data bank (http://www.ncbi.nlm.nih.gov). The 16 S rRNA nucleotide sequence of BEI-18 A was deposited on GenBank with the accession number PZ379208.

### *Streptomyces* sp. BEI-18 A cultivation

The cultivation medium was formulated with 10 g L^− 1^ yeast extract, 5 g L^− 1^ dextrose, 2 g L^− 1^ soybean extract, 1 g L^− 1^ sodium chloride, and 0.2 g L^− 1^ ammonium sulfate, distributed in three Erlenmeyer flasks containing 150 mL of working volume each. The pH was adjusted to 7.0 (± 0.5), and the medium was sterilized at 121 °C for 30 min. Under a laminar flow hood, a loop of the *Streptomyces* sp. BEI-18 A isolate was transferred to a tube containing 0.85% saline solution (NaCl 0.85%) and 0.1% Tween 80, and then homogenized using a tube vortex mixer. An aliquot corresponding to 5% (v/v) of this microbial suspension was inoculated into each Erlenmeyer flask. After inoculation, the flasks were incubated on an orbital shaker (Solab, SL-222) at 160 rpm for 48 h.

For inoculum scale-up, the same cultivation medium was prepared for bioreactors with a 10 L working volume, adjusted to pH 7.0, and sterilized at 121 °C for 30 min. Under aseptic conditions, 48-hour Erlenmeyer flask cultures were used to inoculate the bioreactor at 4.5% (450 mL, v/v). The submerged fermentation was carried out in the bioreactor for 48 h at 28 °C, with continuous aeration at 10 L min^− 1^.

### Procedure for the production of the potential soil conditioner

**Fig. 2:**
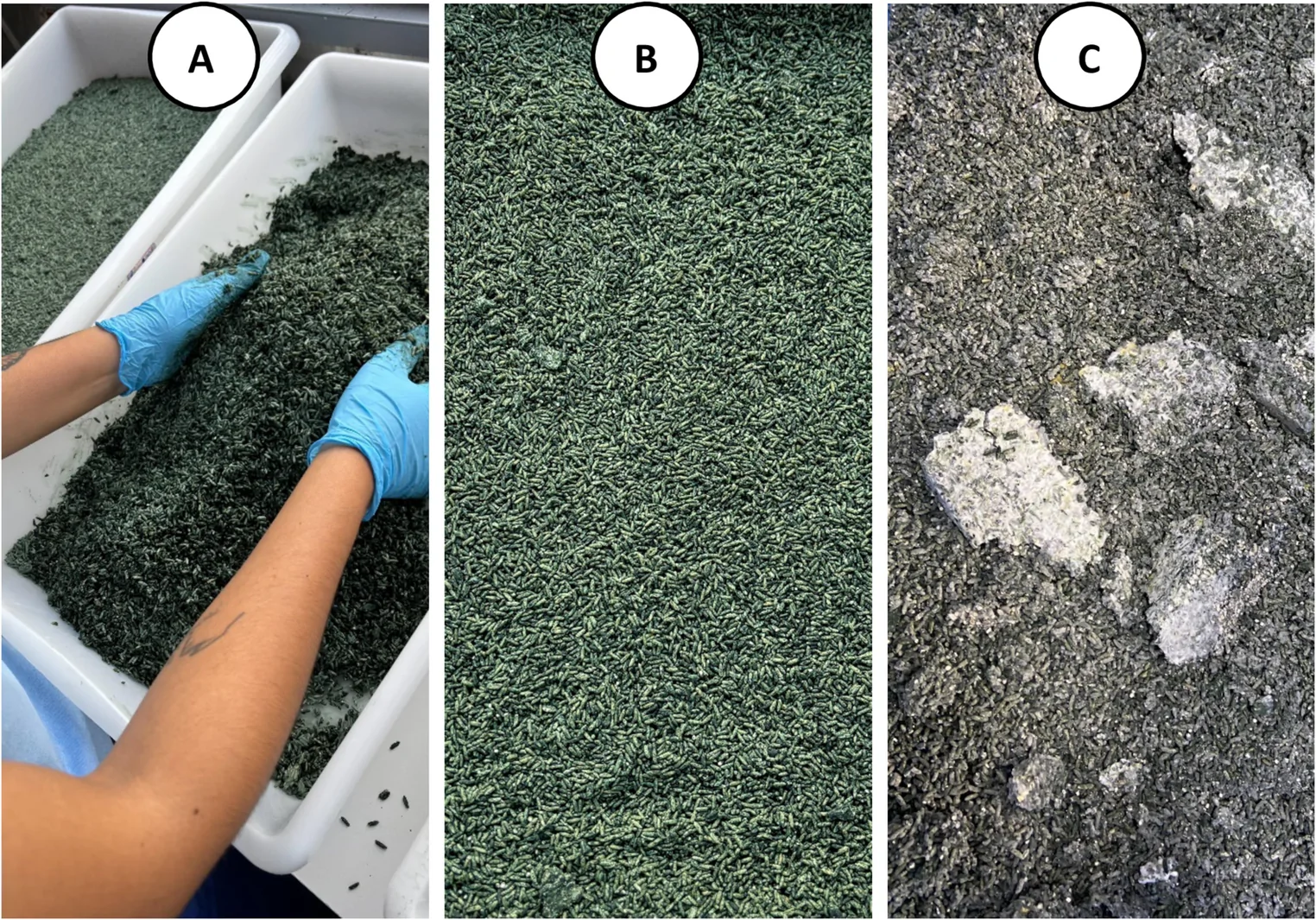
Mixture prepared for the production of the potential soil conditioner: (**A**) formulation stage, (**B**) beginning of the process, and (**C**) end of the process

RWTP was distributed in plastic trays, forming a layer approximately 10 cm thick. Four trays were prepared, each containing 5 kg of RWTP, to which 20% of *Streptomyces* sp. BEI-18 A inoculum and 10% of a solution containing 40 g L^− 1^ sugar and 20 g L^− 1^ sodium chloride were added. The material was manually homogenized prior to fermentation. The trays were covered with brown paper to minimize external contamination and maintained for seven days in a closed room under controlled temperature conditions (25 °C) during solid-state fermentation. No additional mixing was performed during the fermentation period in order to reduce contamination risks. Parameters such as water activity, internal substrate temperature, pH, and moisture content were not monitored throughout the process. The four trays were used as production units for obtaining sufficient material for subsequent analyses. After fermentation, the material from all trays was homogenized to obtain a composite sample used for physicochemical and biological evaluations. Figure 2 shows: (A) the mixture of RWTP, inoculum, and solution; (B) the initiation of SSF; and (C) the product obtained after seven days of fermentation.

### Procedure for Classification as a Soil Conditioner

The potential biological soil conditioner obtained from the cultivation of the actinobacterium *Streptomyces* sp. BEI-18 A using RWTP as substrate was evaluated for its physicochemical and biological properties to ensure compliance with the Normative Instruction SDA No. 35/2006 [40]. The analyses were carried out in the company’s own laboratory (BeiLab), which is duly registered with the Ministry of Agriculture, Livestock, and Supply (MAPA) for conducting analyses of organic fertilizers, substrates, organomineral products, inoculants, and related materials.

According to Normative Instruction SDA No. 35/2006, the biological properties of soil conditioners must be reported in the registration process whenever they can be quantitatively measured [40]. For this evaluation, the following parameters were determined: colony-forming unit (CFU) count, enzymatic activity, and respirometry. CFU determination was performed by weighing 10 g of the product, which were transferred to Erlenmeyer flasks containing 90 mL of a previously autoclaved 0.85% saline solution (NaCl 0.85%) with 0.1% (w/v) Tween 80. The samples were shaken on an orbital shaker (Solab, SL-222) for 20 min at 180 rpm and subsequently subjected to tenfold serial dilution (Brazil, 2010) and plating on ISP-2 (*International Streptomyces Project*) agar in Petri dishes. Only colonies presenting morphological characteristics compatible with *Streptomyces* sp. BEI-18 A were considered for CFU quantification. Colony identification was based on macroscopic morphology and Gram staining. No selective agents or molecular confirmation procedures were used after plating. Arylsulfatase and β-glucosidase activities were determined according to the BioAS protocol based on Tabatabai (1994) [53]. For arylsulfatase, p-nitrophenyl sulfate (PNS 0.05 M) was used as substrate in acetate buffer (0.5 M, pH 5.8), while β-glucosidase activity was determined using p-nitrophenyl-β-D-glucopyranoside (PNG 0.025 M) in modified universal buffer (MUB, pH 6.0) [53]. Samples were incubated at 37 °C for 1 h, and enzymatic activity was quantified by UV-Vis spectrophotometry at 410 nm for arylsulfatase and 420 nm for β-glucosidase based on the release of p-nitrophenol [53]. Calibration curves were prepared using standard p-nitrophenol solutions ranging from 0 to 250 µg p-nitrophenol [53]. Control samples without substrate addition during incubation were included and subtracted from sample readings prior to calculation of enzymatic activity [53]. Results were expressed as µg p-nitrophenol g⁻¹ dry soil h⁻¹ [53]. Table 1 presents the analyses required for the registration of soil conditioners, as well as the methodologies employed for each physicalchemical and biological test.

**Table 1:** Methodology of the physical-chemical and biological tests for classification of the material as a soil conditioner

Parameters	Method
Cation Exchange Capacity (CEC)	SDA/MAPA - IN 17/2007 [50]
Water Retention Capacity at 10 cm (WRC_10_)	SDA/MAPA - IN 17/2007 [50] and IN 31/2008 [51]
Colony-Forming Units (CFU)	SDA/MAPA - IN 30/2010 [52]
Enzymatic Activity	UV-Vis Spectrophotometry [53]
Respirometry	Decesaro et al. [54]

### Respirometry assay

The measurement of the respiratory rate, or microbial activity, is determined by the evolution of carbon dioxide (CO₂) resulting from the respiration of microorganisms during the oxidation of organic compounds. To quantify the CO₂ produced by microbial activity in the potential biological soil conditioner, a respirometry assay was performed as described by Decesaro et al. [54]. The treatments used are presented in Table 2 and were conducted in biological duplicate using independent experimental units. The same soil batch was used for all treatments in order to minimize variability associated with soil properties and native microbiota. For each treatment, 200 g of sample were placed in hermetically sealed flasks and incubated at 20 °C under BOD incubator conditions for 35 days. No additional moisture adjustment was performed during the incubation period.

**Table 2:** Treatments performed in the respirometry test

Treatments	Composition
Blank (TB)	Without soil
Control Treatment (CT)	200 g of soil
Treatment 2.5% (T2.5)	195 g of soil + 5 g of potential biological soil conditioner
Treatment 5% (T5)	190 g of soil + 10 g of potential biological soil conditioner
Treatment 10% (T10)	180 g of soil + 20 g of potential biological soil conditioner

Inside each flask, 30 mL of 2 mol L⁻¹ NaOH solution were added, which was replaced every two to three days after sample collection. For titration, 10 mL of the NaOH solution were withdrawn and mixed with 10 mL of 0.2 mol L⁻¹ BaCl₂ solution and two drops of 1% phenolphthalein, used as an indicator (color change from pink to colorless). The quantification of the CO₂ produced was performed by titration with 0.5 mol L⁻¹ HCl, as shown in Eq. 1.

1 $$\:C-{CO}_{2\:\left(mg\right)}=\left(B-V\right)\times\:M\times\:f\times\:6\times\:\frac{V1}{V2}$$

Where: B – volume of HCl used in the blank (mL); V – volume of HCl used in the sample (mL); M – actual concentration of HCl (mol L⁻¹); 6 – atomic mass of carbon (12 u) divided by the number of moles of CO₂ reacting with NaOH; V1 – volume of NaOH used for CO₂ capture (mL); V2 – volume of NaOH used for titration (mL); f – correction factor for HCl.

### Evaluation of the potential plant growth-promoting properties of the material

The potential biological soil conditioner obtained through SSF was evaluated for its ability to promote plant growth using pot experiments. The experimental design included four treatments, each conducted in quadruplicate using independent pots as biological replicates, as presented in Table 3.

**Table 3:** Treatments applied in the seedling assay

Treatments	Composition
Control Treatment (CT)	250 g soil
Treatment 2.5% (T2.5)	244 g soil + 6 g potential biological soil conditioner
Treatment 5% (T5)	238 g soil + 12 g potential biological soil conditioner
Treatment 10% (T10)	226 g soil + 24 g potential biological soil conditioner

The application rates were selected to provide a gradient of biological soil conditioner incorporation into the soil, enabling the evaluation of dose-dependent responses under preliminary pot assay conditions. The selected concentrations were exploratory and were not intended to represent agronomic recommendations or regulatory application rates. Maize (*Zea mays* L.) was selected for the evaluation as it was in an appropriate planting period. In each pot containing 250 g of soil, three seeds of hybrid maize (K9606 VIP3) were sown. The soil used in the treatments was collected from Linha 19 (29°19′54.97″S; 54°29′46.71″W), municipality of Carlos Barbosa/RS, Brazil, following the ABNT NBR 6457:2016 standard - Soil sampling: preparation of samples for testing and determination of moisture content [55], and was classified as Nitosol. The pots were arranged in a randomized design to minimize variations arising from environmental factors such as sunlight exposure, shading, and precipitation. Irrigation was performed manually according to plant demand and visual soil moisture conditions in order to maintain soil moisture close to field capacity. The applied water volume was not quantified, but irrigation was standardized across treatments to minimize water-related variability.

The experiment lasted 21 days in order to evaluate the initial effects of the potential biological soil conditioner during early maize development. During this period, the following plant parameters were assessed: morphometry (shoot height and stem diameter), root dry mass, shoot dry mass, and the Dickson Quality Index (DQI). Height was measured using a graduated ruler, while stem diameter was determined with a caliper. For dry mass determination, roots and shoots were dried in an oven at 65 °C until constant weight was achieved. The DQI was calculated according to Dickson et al. [56], using the following Eq. 2:

2 $$\:DQI=\:\frac{SDM}{\left(\frac{H}{SD}\right)+\left(\frac{{SDM}_{shoot}}{{SDM}_{root}}\right)}$$

Where: SDM = total seedling dry mass (g); H = shoot height (cm); SD = stem diameter (mm); SDMₛₕₒₒₜ = shoot dry mass (g); SDMrₒₒₜ = root dry mass (g)

**Fig. 3:**
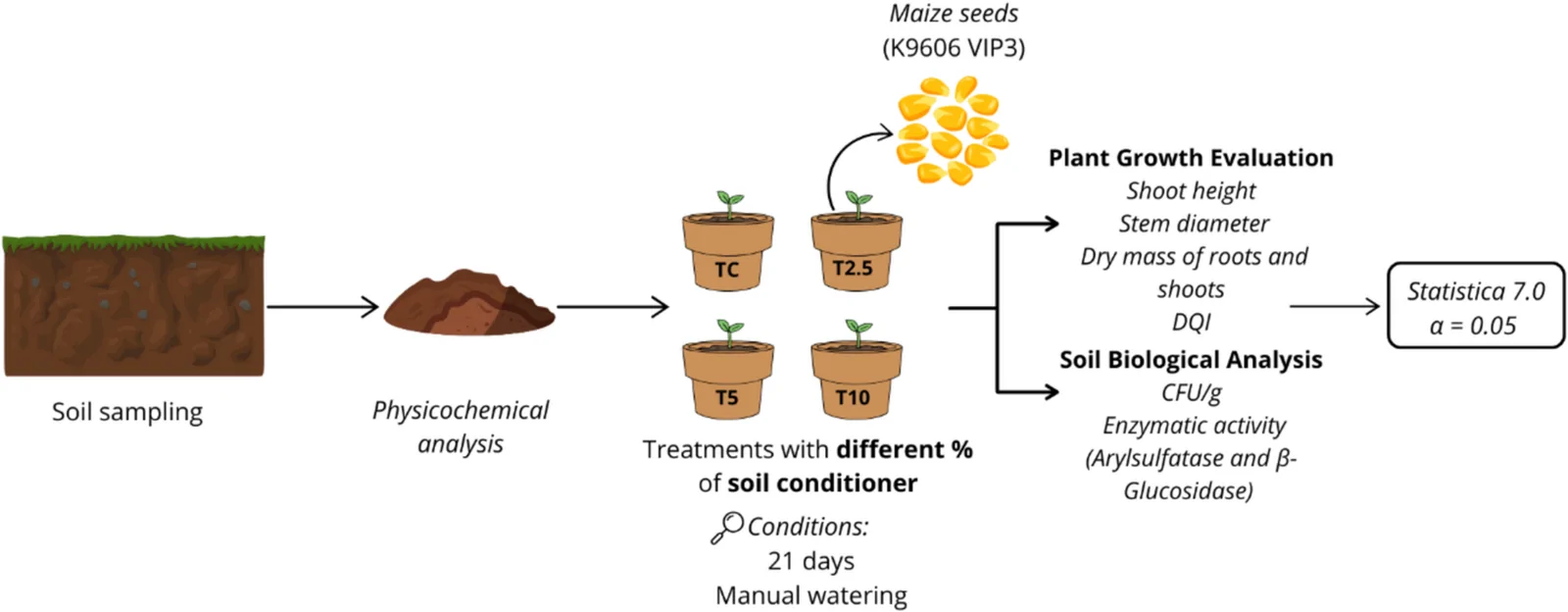
Flowchart of initial maize growth promotion assessment using the potential biological soil conditioner

After the completion of the experiment, the soil from each treatment was evaluated for enzymatic activity and colony-forming units (CFU g⁻¹) in the treated soils. The sequence of experimental steps, from soil collection to statistical analyses, is illustrated in Fig. 3.

### Statistical analysis

Experimental data were subjected to analysis of variance (ANOVA), with differences among treatments evaluated using Tukey’s test at a 5% significance level (α = 0.05). The statistical test was performed on the final cumulative values. All statistical analyses were performed using Statistica 7.0 software (StatSoft, USA).

## Results and discussion

### Isolate identification

**Fig. 4:**
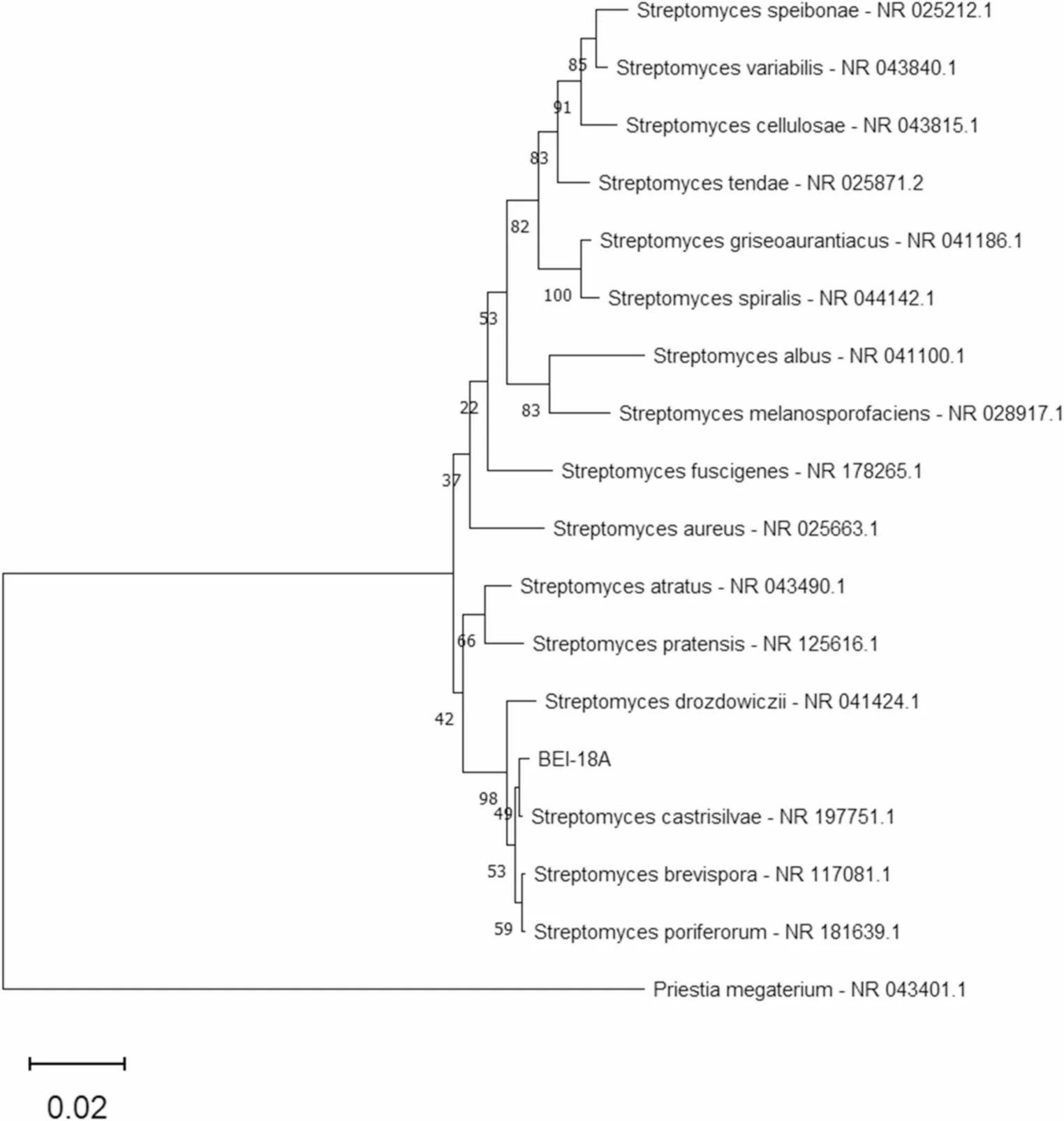
Phylogenetic tree based on 16 S rRNA gene sequences of isolate BEI-18 A and related *Streptomyces* species. Legend: Bootstrap values were calculated from 1000 replicates. The tree was rooted with *Priestia megaterium*

The BEI-18 A isolate was identified as *Streptomyces* sp. (GenBank accession number: PZ379208), with the closest reference species being *Streptomyces castrisilvae* NR197751.1 (99.22%), *Streptomyces poriferorum* NR181639.1 (99.06%), and *Streptomyces brevispora* NR_117081.1 (98.91%) (Fig. 4). Additional information regarding the functional characterization of the isolate can be found in Nazari et al. [19].

### Evaluation of the potential biological soil conditioner

According to Normative Instruction SDA No. 35/2006, soil conditioners in Brazil may be classified based on physicochemical or biological properties, depending on the product characteristics and intended classification [40]. For physicochemical classification, minimum thresholds are established for parameters such as cation exchange capacity (CEC) and water retention capacity (WRC_10_). In contrast, biological soil conditioners do not require minimum reference values, however, their biological properties must be quantitatively measurable. Therefore, in the present study, physicochemical parameters required by the regulation were evaluated together with measurable biological properties, including microbial concentration (CFU g⁻¹) and enzymatic activity.

#### Physicochemical evaluation

Table 4 presents the results of the physicochemical analyses performed on the potential biological soil conditioner.

**Table 4:** Physicochemical results of the *Streptomyces*-based potential biological soil conditioner

Parameters	Result	Comparison with the Normative Instruction SDA No. 35/2006 [40]
CEC	59.3 mmol_c_ kg^− 1^	Minimum of 200 mmol_c_ kg^− 1^
WRC_10_	89.1%	Minimum of 60%

Analyses were performed on a composite sample obtained from pooled fermentation trays, with results expressed as a single analytical value

The product presented a CEC of 59.3 mmol_c_ kg^− 1^, below the minimum value required by Normative Instruction SDA No. 35/2006 for classification as a physicochemical soil conditioner. In contrast, WRC_10_ reached 89.1%, exceeding the minimum required value. CEC represents the total amount of cations that colloids, humic substances, and iron and aluminum oxides can adsorb [57]. These materials generally exhibit a higher number of negative charges, favoring cation adsorption. However, some sites may present positive charges, allowing anion adsorption, a phenomenon more commonly observed in iron and aluminum oxides [57]. According to Ronquim [57], optimal plant development requires higher concentrations of cations such as Ca²⁺, Mg²⁺, and K⁺. Conversely, when H⁺ and Al³⁺ predominate, nutrient availability is impaired. The observed value suggests that the potential biological soil conditioner has a limited capacity to retain cations in exchangeable form. Considering that pH (hydrogen potential) reflects the concentration of H⁺ ions in the material, the value of 4.23 obtained for the product partially explains the low CEC, due to the greater presence of H⁺, which directly influences this parameter. According to Normative Instruction SDA No. 35/2006, the minimum CEC value required for classification as a physicochemical conditioner is 200 mmolc/kg, a value not achieved in this study [40].

WRC_10_, a parameter that directly influences water availability to plants, showed satisfactory results, exceeding 60%. The organic carbon content, which is strongly related to WRC_10_, was 39.8%, which may be associated with higher porosity and water retention capacity. The presence of humic substances contributes to the formation of stable aggregates and macropores, favoring water infiltration, aeration, and resistance to erosion [57–58]. Several studies have documented this relationship, emphasizing that the formation of stable aggregates enhances structural stability and erosion resistance [59–62]. In addition, high amounts of soil organic matter, associated with improved structure, contribute to increased enzymatic activity, demonstrating a direct connection between these parameters [63–64]. The WRC_10_ is particularly relevant under water stress conditions, as it provides the soil with greater drought tolerance, supporting plant development even in adverse scenarios [65].

Therefore, although the material showed satisfactory water retention capacity, its physicochemical performance was only partial, since the minimum CEC requirement established by Normative Instruction SDA No. 35/2006 was not achieved. Consequently, the product could not be classified as a physicochemical soil conditioner according to the Brazilian regulation [40].

#### Bacterial concentration and enzymatic activities of the potential biological soil conditioner

Table 5 presents the results of the biological analyses conducted to characterize the potential biological soil conditioner.

**Table 5:** Results of Biological Analyses of the *Streptomyces*-Based Potential Biological Soil Conditioner

Parameters	Result
*Streptomyces* sp. BEI-18 A concentration^*^	7.65 ± 0.05 Log CFU g^− 1^ (4.5 × 10⁷ CFU g^− 1^)
Arylsulfatase enzymatic activity	28.79 ± 5.32 µg p-nitrophenol/g soil/h
β-Glucosidase enzymatic activity	846.79 ± 26.55 µg p-nitrophenol/g soil/h

Analyses were performed in duplicate on a composite sample obtained from pooled fermentation trays, and the results are expressed as mean values ± standard deviation. ^*^Quantification of *Streptomyces* sp. BEI-18 A considered only colonies exhibiting morphological characteristics consistent with the isolate, based on colony macro- and micromorphology

The initial inoculum concentration of *Streptomyces* sp. BEI-18 A was 7.81 Log CFU g^− 1^ (6.5 × 10⁷ CFU g⁻¹), and after seven days of solid-state fermentation, the potential biological soil conditioner exhibited 7.65 ± 0.05 Log CFU g^− 1^ (4.5 × 10⁷ CFU g⁻¹). This reduction could potentially be related to competitive interactions within the substrate, including the presence of residual *Trichoderma* structures remaining after conidia separation [25, 66], although this hypothesis was not specifically evaluated in the present study. Nevertheless, the final count remained within the expected range for similar systems for a *Streptomyces*-based product. Previous studies also indicate that *Streptomyces* can maintain viable populations in competitive environments. Marian et al. [67], when applying *Streptomyces* sp. MBFA-172 for anthracnose control in strawberry, observed values of 1.0 × 10⁶ CFU g⁻¹ in mature leaves one week after treatment, 3.4 × 10⁵ CFU g⁻¹ in young leaves three weeks after, and 3.0 × 10⁵ CFU g⁻¹ in the crown after one week.

β-Glucosidase activity reached 846.79 µg p-nitrophenol g⁻¹ soil h⁻¹, whereas arylsulfatase activity reached 28.79 µg p-nitrophenol g⁻¹ soil h⁻¹. Both enzymes participate in nutrient cycling and organic matter decomposition and are commonly used as indicators of soil biological activity [68]. β-Glucosidase breaks down cellulose into glucose, increasing carbon availability [69], while arylsulfatase converts sulfate esters into plant-assimilable sulfate [70–71]. The literature reports that in *Streptomyces*, arylsulfatase can be associated with the cell membrane, stimulated by a specific substrate, regulated independently of sulfur demand, or present intracellularly, being induced under low sulfate availability [72–73]. In turn, β-glucosidase is predominantly produced by actinobacteria during composting processes, with abundance ranging from 61.7% to 93.3% depending on the substrate and additives such as biochar [74–75]. Long-term experiments have reported that agro-industrial residues may enhance soil enzymatic activity and improve agronomic parameters. Tejada and Benítez [76] observed increases of up to 42.5% in β-glucosidase activity and 15.8% in arylsulfatase activity in soils treated with green forage vermicompost compared to municipal solid waste, resulting in yield gains of up to 35.5% in olive trees.

To date, no reference values for enzymatic activity in biological soil conditioners or similar products are available, with the only benchmarks being enzyme levels present in soil [68]. Nevertheless, the results demonstrated substantial β-glucosidase activity, suggesting microbial metabolic activity associated with carbon cycling and organic matter decomposition in the formulated biological soil conditioner. This result may be related to the predominance of actinobacteria, which are frequently reported as important producers of β-glucosidase during organic matter degradation processes [74–75]. According to Normative Instruction SDA No. 35/2006 [40], both cell concentration (CFU g⁻¹) and enzymatic activity may be quantified and reported in the registration process of biological soil conditioners.

#### Respirometry

**Fig. 5:**
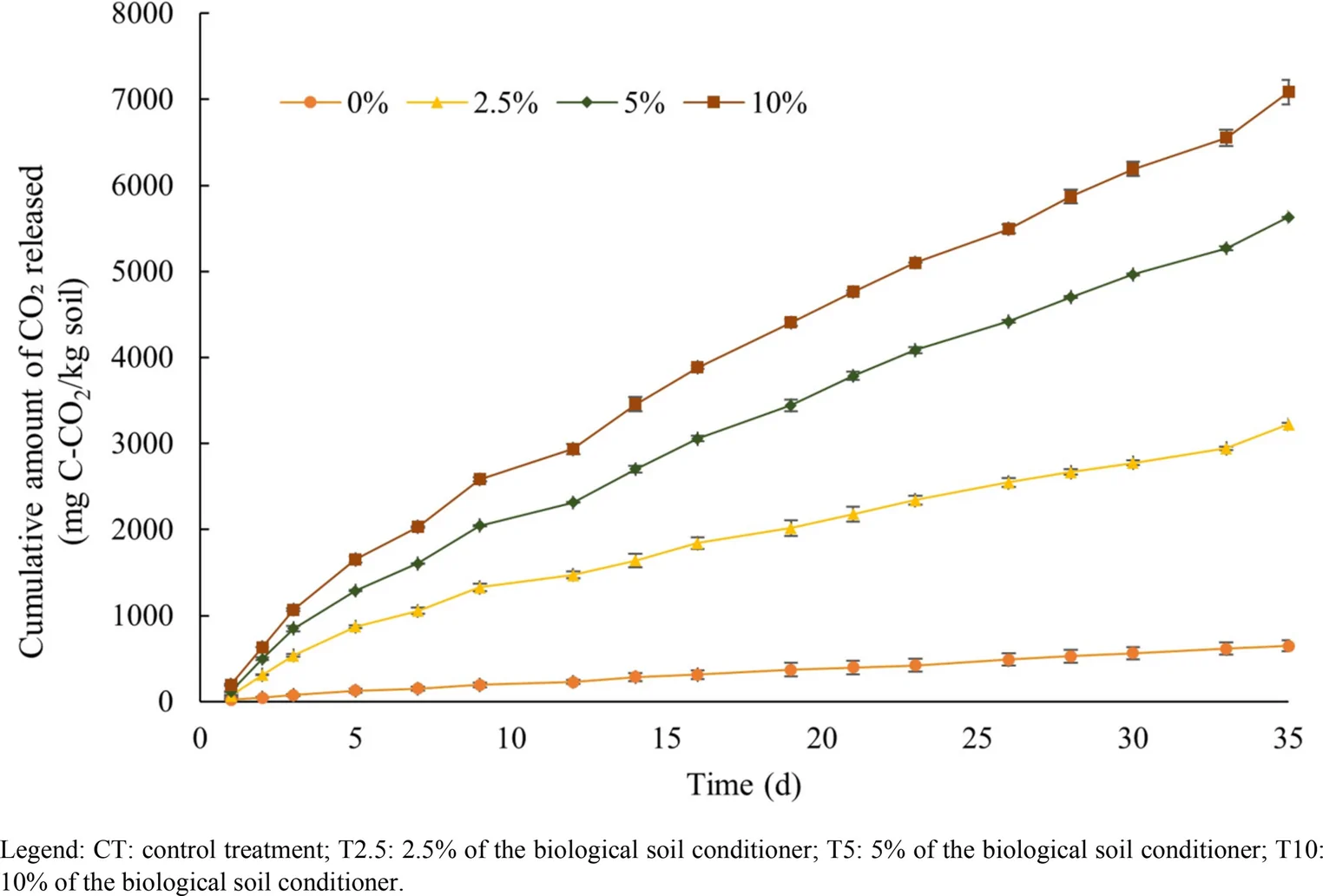
Cumulative amount of CO₂ released over time. Legend: CT: control treatment; T2.5: 2.5% of the biological soil conditioner; T5: 5% of the biological soil conditioner; T10: 10% of the biological soil conditioner

To estimate the microbial activity of the potential biological soil conditioner and provide quantitative data for its registration, a respirometry analysis was conducted (Fig. 5). According to Decesaro et al. [54], during the degradation of organic compounds, microorganisms in the medium release CO₂ and H₂O, with CO₂ considered an important indicator of microbial activity.

The graph shows the cumulative amount of CO₂ released over the 35-day experiment, highlighting significant differences between treatments. The statistical test was performed on the final cumulative values. On day 5, the control recorded 125.87 mg C-CO₂ kg⁻¹ soil, whereas treatment T10 reached 1,651.35 mg C-CO₂ kg⁻¹ soil, representing an increase of approximately 13.12-fold in CO₂ release, suggesting greater microbial metabolic activity. Moreover, the potential biological soil conditioner itself may have served as a substrate source, promoting the proliferation and metabolism of microorganisms already present in the soil, resulting in higher CO₂ production. Throughout the experimental period, a gradual increase in CO₂ release was observed in treatments T2.5, T5, and T10, indicating sustained microbial metabolic activity throughout the experimental period. At the end of the 35-day period, the control recorded 647.35 mg C-CO₂ kg⁻¹ of soil, whereas treatments T2.5, T5, and T10 reached 3.221.78, 5.628.37 and 7.081.91 mg C-CO₂ kg⁻¹ of soil, respectively, with the latter being 10.94 times higher than the control (*p* < 0.05). These results indicate that the biological soil conditioner stimulated microbial metabolic activity in the soil, supporting its biological characterization based on measurable microbial parameters.

Luan et al. [77] investigated the influence of different mechanisms associated with soil respiration, including the addition of organic matter, microbial community composition, and enzymatic activity. Their results indicated that increasing the input of organic matter significantly enhances the respiration rate, particularly due to the action of hydrolytic enzymes such as β-glucosidase, whose activity showed a positive correlation with this parameter. Microbial community composition was also identified as a key factor, as it directly influences enzymatic activity. Thus, the authors concluded that soil microbiology exerts a positive effect on enzymatic activity levels, consequently promoting increased soil respiration. In this way, changes in soil organic composition may intensify microbial respiration and organic matter mineralization processes, influencing carbon turnover in the soil. Increased microbial activity is associated with organic matter decomposition and nutrient cycling processes in soil systems [68, 78]. However, elevated CO₂ release should be interpreted cautiously, as higher respiration rates may also reflect rapid mineralization and loss of readily available organic carbon rather than long-term carbon stabilization. Therefore, the respirometry analysis performed in the present study should be interpreted primarily as an indicator of microbial metabolic activity associated with the application of the biological soil conditioner.

### Evaluation of the performance of the biological soil conditioner as a plant growth promoter

Table 6 presents the results of early maize growth in soil treated or untreated with the actinobacteria-based biological soil conditioner.

**Table 6:** Morphometric results of early maize growth

Treatments	SH (cm)	SD (mm)	RDM (g)	SDM (g)	DQI
CT	15.96 ± 1.91^A^	4.12 ± 0.31^A^	0.0934 ± 0.02^A^	0.0795 ± 0.02^A^	0.0344 ± 0.007^A^
T2.5	15.00 ± 1.04^A^	4.25 ± 0.35^A^	0.0740 ± 0.003^AB^	0.0773 ± 0.008^A^	0.0336 ± 0.003^A^
T5	14.36 ± 1.03^A^	3.8 ± 0.24^A^	0.0676 ± 0.004^AB^	0.0642 ± 0.007^A^	0.0273 ± 0.002^A^
T10	13.71 ± 0.86^A^	3.78 ± 0.15^A^	0.0598 ± 0.004^B^	0.0624 ± 0.005^A^	0.0266 ± 0.002^A^

*CT *control treatment; T2.5: 2.5% of the biological soil conditioner, *T5 *5% of the biological soil conditioner, *T10 *10% of the biological soil conditioner, *SH *shoot height, *SD *stem diameter, *RDM *root dry mass, *SDM *shoot dry mass, *DQI *Dickson Quality Index. Values represent the mean ± standard deviation of four biological replicates (*n* = 4; independent pots). Different letters within a column indicate significant differences among means according to Tukey’s test (*p* < 0.05)

For the parameters of shoot height, stem diameter, and shoot dry mass, no significant differences were observed among treatments and the control (*p* > 0.05). However, regarding root dry mass, the T10 treatment showed lower performance, while T2.5 and T5 did not differ from the control. No clear phytotoxic effects were observed at the 2.5% and 5% application rates, whereas the 10% treatment negatively affected root dry mass under the evaluated conditions. Table 7 presents selected chemical parameters of the soil used in the maize assay and their comparison with literature reference values.

**Table 7:** Chemical analysis results of the soil

Parameter	Sample value	Liming and fertilization manual RS/SC [79]	
		Reference value	Interpretation
pH	5.9	6.0	Ideal
CEC	22.6 cmolc/dm³	15.1–30.0 cmolc/dm³	High
Base Saturation (V)	86.7%	-	-
Clay Content	34%	21–40%	Class 3
Phosphorus (P)	71.2 mg/dm³	> 36.0 mg/dm³	Very high
Potassium (K)	319 mg/dm³	> 240 mg/dm³	Very high

Analyses were performed on a composite sample obtained from pooled fermentation trays, with results expressed as a single analytical value

The limited agronomic response observed in the maize assay is likely associated with the high fertility of the soil used, which presented near-ideal pH, high CEC, very high phosphorus levels, and very high potassium availability. These conditions may have reduced the ability to detect additional plant responses associated with the application of the biological soil conditioner during the experimental period. According to the Liming and Fertilization Manual for the States of Rio Grande do Sul and Santa Catarina [79], the ideal pH for maize cultivation is 6.0. The pH measured in the experimental soil was 5.9, which is consistent with this recommendation and supports satisfactory plant development. The phosphorus (P) content, evaluated according to the clay content, was classified as very high for grain crops, as was potassium (K), whose interpretation was based on the soil cation exchange capacity (CEC). Both nutrients are essential for plant development: phosphorus stimulates root growth, while potassium is involved in enzymatic activation, stomatal regulation, water management, and photosynthesis [82]. The base saturation (V), which expresses the percentage of the CEC occupied by exchangeable cations (Ca²⁺, Mg²⁺, and K⁺), combined with the high CEC, confirms the high nutrient availability. According to Ronquim [57], fertile soils present V ≥ 50%. Therefore, the soil used exhibited characteristics suitable for high productivity and plant vigor.

The Soil Bioanalysis (BioAS) technology, developed by Embrapa in 2020, assesses the activity of the enzymes arylsulfatase and β-glucosidase, which are associated with the sulfur and carbon cycles, respectively. These enzymes act as bioindicators because they participate in the decomposition and formation of organic matter, influencing soil structure, water dynamics, carbon sequestration, and greenhouse gas mitigation [78]. Reference values for arylsulfatase and β-glucosidase in clayey Oxisols from the Cerrado biome, at a depth of 0–10 cm and under post-harvest conditions, according to the FERTBIO concept, are presented in Table 8 [68]. It is important to note that these reference values were obtained from long-term experiments conducted in the Cerrado region, and, to date, there are no specific tables for other regions of the country. The soil from the municipality of Carlos Barbosa, where the samples for this study were collected, is classified as a Typic Kandiudalf [80], with a clayey texture confirmed by physical analysis, which characterized it as Type 3, corresponding to clayey soils with a clay content equal to or greater than 35%. Ongoing studies aim to develop regional reference tables that consider different management systems [68]. Table 8 presents the results of the soil biological parameters, both for samples inoculated and non-inoculated with the biological soil conditioner, as well as their classification according to the interpretation classes established by Embrapa.

**Table 8:** Biological Parameters of Soil Samples Inoculated or Non-Inoculated with the Biological Soil Conditioner and Comparison with Embrapa’s Interpretation Classes

Treatment	Arylsulfatase (µg *p*-nitrophenol g⁻¹ soil h⁻¹)	β-glucosidase (µg *p*-nitrophenol g⁻¹ soil h⁻¹)	Arylsulfatase	β-glucosidase	Microbial concentration (Log CFU g⁻¹)
CT	168.37 ± 7.91^B^	75.53 ± 2.81^D^	Adequate	Moderate	7.01 ± 0.10^B^
T2.5	203.48 ± 0.49^A^	128.41 ± 6.41^C^	Adequate	Adequate	8.19 ± 0.18^A^
T5	187.24 ± 16.25^AB^	181.46 ± 6.00^B^	Adequate	Adequate	8.27 ± 0.16^A^
T10	189.46 ± 5.88^AB^	270.26 ± 4.17^A^	Adequate	Adequate	8.47 ± 0.21^A^

*CT *control treatment, *T2.5 *soil treated with 2.5% biological soil conditioner, *T5* 5% biological soil conditioner, *T10* 10% biological soil conditioner; Values represent the mean ± standard deviation of duplicate analyses (*n* = 2). Different letters within a column indicate significant differences among means according to Tukey’s test (*p* < 0.05)

All treatments showed adequate results for arylsulfatase activity. However, treatment T2.5 exhibited a significant increase compared to the control (*p* < 0.05), indicating stimulation of enzymatic activity associated with sulfur cycling. For β-glucosidase, enzymatic activity in the control was classified as moderate and progressively increased with the application of the biological soil conditioner, reaching adequate levels according to the adopted interpretation classes. In all inoculated treatments, a significant and progressive increase in β-glucosidase activity was observed (*p* < 0.05). This increase may be associated with the role of actinobacteria in organic matter decomposition and carbon cycling processes in soil systems [29, 37, 38].

The microbial concentration in the soil (Log CFU g⁻¹) increased significantly following inoculation (*p* < 0.05), with the inoculated treatments exhibiting statistically higher microbial abundance than the non-inoculated control soil (Table 8). The increase in enzymatic activity may be associated with greater microbial activity, since enzymes are produced by microorganisms [77, 81]. Inoculation with the biological soil conditioner increased soil biological parameters, including microbial abundance and enzymatic activity, supporting its characterization based on measurable biological properties. However, these biological responses were not accompanied by significant improvements in most maize growth parameters during the experimental period, and treatment T10 resulted in reduced root dry mass compared to the control. The limited plant growth response may be associated with the high fertility conditions of the soil used and the short evaluation period, which may have restricted the detection of additional plant responses associated with the biological soil conditioner. Therefore, further studies under low-fertility or degraded soil conditions and over longer cultivation periods are necessary to better assess its agronomic performance.

Although the increases in microbial and enzymatic parameters support the biological activity of the formulated product, the absence of additional controls, such as non-inoculated RWTP, sterilized substrate, or commercial soil conditioners, limits the ability to distinguish the individual contributions of the organic substrate, residual *Trichoderma* structures, and *Streptomyces* sp. BEI-18 A to the observed effects. Nevertheless, the present study provides an initial evaluation of the biological performance of the final product obtained through the proposed fermentation process, demonstrating its capacity to stimulate soil microbial and enzymatic activity under the evaluated conditions. Future studies including additional controls and microbiological characterization of the substrate may contribute to a more detailed understanding of the mechanisms involved.

## Conclusion

Based on the results obtained, a biological soil conditioner was obtained based on the actinobacterium *Streptomyces* cultivated on rice waste from the production of the fungus *Trichoderma asperelloides*. According to the Normative Instruction SDA No. 35/2006, the product is classified as Class A - Biological, since it promoted an increase in microbial abundance, enzymatic activity, and microbial activity, as measured by CO₂ release. The biological soil conditioner presented measurable biological parameters, as evidenced by the CFU g⁻¹ count and the elevated β-glucosidase activity, an enzyme essential for the carbon cycle, indicating a significant contribution to fundamental biological processes in the soil. The water retention capacity (WRC_10_), a physicochemical parameter evaluated, proved adequate, suggesting the product’s potential to enhance soil resilience under extreme conditions, such as drought periods.

In the maize pot experiments, the application of the biological soil conditioner increased soil biological parameters, as evidenced by higher microbial abundance and enhanced β-glucosidase and arylsulfatase activities compared to the control. Notably, the β-glucosidase activity classification increased from moderate to adequate. The respirometry analysis also showed a significant rise in the cumulative amount of CO₂ released, evidencing high microbial activity. The product may contribute to sustainable soil-management strategies, but further field studies are required to confirm its agronomic and environmental benefits. In addition to providing a solution for the disposal of an industrial byproduct, the technology contributes to the reuse of agro-industrial wastes, aligning with several United Nations Sustainable Development Goals (SDGs), particularly SDG 2 (Zero Hunger), SDG 9 (Industry, Innovation, and Infrastructure), SDG 12 (Responsible Consumption and Production), and SDG 15 (Life on Land).

## Data Availability

The manuscript has no associated data.
